# Cancer Vaccines: Recent Insights and Future Directions

**DOI:** 10.3390/ijms252011256

**Published:** 2024-10-19

**Authors:** Aretia-Teodora Malacopol, Peter Johannes Holst

**Affiliations:** 1 HERVOLUTION Therapeutics, Copenhagen Bio Science (COBIS), 215 Nordre Fasanvej, DK2200 Copenhagen, Denmark; tmalacopol@hervolutiontx.com; 2 Department of Biomedical Sciences, The Panum Institute, University of Copenhagen, DK2200 Copenhagen, Denmark

**Keywords:** cancer vaccines, immune system, tumor-specific antigens, neoantigens, checkpoint inhibitors, HERVs

## Abstract

The field of cancer immunotherapy has seen incredible advancements in the past decades. mRNA-based cancer vaccines generating de novo T cell responses, particularly against tumor-specific antigens (TSAs), have demonstrated promising clinical outcomes and overcome diverse challenges. Despite the high potential of neoantigens to provide personalized immunotherapies through their tumor specificity and immunogenicity, challenges related to the scarcity of immunogenic neoepitopes have prompted continuous research towards finding new tumor-associated antigens (TAAs) and broader therapeutic frameworks, which may now learn from the genuine successes obtained with neoantigens. As an example, human endogenous retroviruses (HERVs) have emerged as potential alternatives to tumor neoantigens due to their high tumoral expression and ability to elicit both T cell reactivity and B cell responses associated with the efficacy of existing immunotherapies. This review aims to assess the status and limitations of TSA-directed mRNA cancer vaccines and the lessons that can be derived from these and checkpoint inhibitor studies to guide TAA vaccine development. We expect that shared B cell, CD4 and CD8 T cell antigen presentation will be key to stimulate continuous T cell expansion and efficacy for tumors that do not contain pre-existing tertiary lymphoid structures. When these structures are present in highly mutated tumors, the current checkpoint-based immunotherapies show efficacy even in immune privileged sites, and vaccines may hold the key to broaden efficacy to more tumor types and stages.

## 1. Introduction

### 1.1. Breakthroughs in the Cancer Vaccine Field

The last 1–2 years have yielded important breakthroughs in cancer vaccination, proving the ability of active immunization with defined recombinant antigens to yield clinically meaningful improvements in patient outcomes. Notably, the three-year Phase 1 follow-up from BioNTech’s neoantigen individualized mRNA vaccine (autogene cevumeran (BNT122, RO7198457)) clinical study for patients after complete resection of pancreatic ductal adenocarcinoma (PDAC) has shown promising results. PDAC is a type of tumor marked by reduced tumor mutational burden and an associated enhanced immunosuppressive tumor microenvironment, with a disease relapse rate and progression after surgery in about 80% of the patient population, placing it in an unmet medical need setting with very poor clinical outcomes. In the study, half of the patients exhibited a persistent and high-amplitude T cell response up to three years after administration, with an associated enlarged T cell receptor neoantigen specificity and ability to recognize multiple ligands [1]. Although this was not a randomized controlled study, six out of eight patients with an immune response remained disease free during the three-year follow-up period, while seven out of eight patients without an immune response to the treatment during the trial showed tumor recurrence. These data, alongside the previously reported high disease relapse and progression rate after surgery, are highly encouraging and suggest that the delay or prevention of pancreatic cancer recurrence is achievable. The data were supplied in the form of a press release from the company, so these results have not been peer-reviewed yet, but they are still suggestive of the capacity of the recombinant vector vaccines to produce an anti-tumoral effect.

Additionally, the fact that this platform, used in the post-surgical context, is able to induce apparent de novo activation of cytotoxic T cells, is also encouraging, as these are intended to be directed against residual occult disease, functioning as a therapeutic adjuvant to the initial treatment. Furthermore, BioNTech has initiated a Phase 2 clinical trial (NCT05968326) following the aforementioned Phase 1 studies (NCT04161755) that is evaluating the safety and tolerability of the neoantigen individualized mRNA vaccine in combination with anti-programmed cell death-ligand 1 (anti-PD-L1) immune checkpoint inhibitor and chemotherapy, the primary goal of the Phase 2 trials being the assessment of disease-free survival. Given that the Phase 1 studies showed a favorable safety profile associated with the therapy, along with robust vaccine-induced T cell responses that may be correlated with the observed delayed PDAC recurrence, particularly due to this tumor type’s characteristics [2], this approach has a high potential, especially for patients in this high unmet medical need category. The therapeutic index of the autogene cevumeran vaccine is currently being evaluated in various solid tumor settings, including surgically resected colorectal cancer and advanced melanoma.

In conceptually related work, Moderna has initiated several clinical trials to assess the safety, efficacy and clinical potential of their neoantigen vaccines. Their mRNA-based neoantigen vaccines have shown remarkable potential in several ongoing clinical trials. One of their most promising candidates is the mRNA-5671 vaccine, which is designed to target Kirsten rat sarcoma viral oncogene homologue (*KRAS*)-mutant colon, pancreatic and lung cancers. The strategy focuses on targeting well-characterized and consistently expressed neoantigens such as *KRAS* driver mutations that have a uniform expression along the disease progression and are found across various cancer types, rather than sporadic, patient-specific epitopes. These mutations occur at specific sites and subsequently drive the development of a few different oncogenic variants, making them suitable targets for vaccine development. This vaccine is currently tested both as a monotherapy or in combination with anti-PD-1/PD-L1 inhibitors in an ongoing Phase 1 clinical trial (NCT03948763) [3].

A leading candidate within the Moderna mRNA-based vaccine platform is the mRNA-4157 (V940) individualized neoantigen vaccine that is currently being tested in a Phase 2b clinical trial for patients with high-risk stage III/IV melanoma. In combination with Merck’s anti-PD-1 immune checkpoint inhibitor KEYTRUDA, this personalized mRNA vaccine has shown significantly positive clinical and statistical outcomes, particularly in recurrence-free survival. It has also demonstrated a substantial reduction in the risk of recurrence or death in patients receiving the combinatorial therapy compared to those receiving only KEYTRUDA [4]. Based on the early clinical trials, the combination of the mRNA vaccine and KEYTRUDA appears to induce a strong cytotoxic T cell response that further enhances the destruction of tumor cells.

These early but meaningful clinical trials in pancreatic cancer and melanoma highlight the transformative potential of the mRNA-based cancer vaccines in the context of the treatment of solid tumors. mRNA vaccines offer the advantage of simultaneously encoding the entire length of the tumor antigens, enabling the presentation of multiple epitopes by the antigen-presenting cells (APCs) without the risk of integration in the host genome [5]. The mRNA cancer vaccines work by delivering synthetic mRNA encoding for the selected tumor antigens into dendritic cells (DCs), often using lipid nanoparticles to protect the mRNA and facilitate its entry into cells. The mRNA is subsequently translated into the relevant antigenic proteins, which are processed and presented on the surface of the DCs. The encoded antigen thus presented activates T cells, initiating in this way a targeted immune response against cancer cells. For a recent review on mRNA used for cancer vaccines, including variations in their design, formulation and properties as compared to other platforms, the reader is referred to Yang and Cui et al. [6]. mRNA vaccines not only offer customizability and ease of production but also have the ability to elicit both humoral and cellular immune responses. Although the humoral immunity has not been demonstrated, shown or studied in mRNA vaccine research due to antigen choice, the vaccines are anticipated to perform well in this context [7]. In addition to mRNA-based strategies, other vaccine platforms are also being explored. Building on the principles of mRNA-based neoantigen vaccines, Nouscom has developed viral-vector-based personalized vaccination approaches as an alternative platform for targeting multiple neoantigens with the main goal of inducing a broader antitumor T cell response. These approaches utilize adenovirus and poxvirus prime–boost immunization for targeting patient-specific and shared mutations and have shown encouraging results in initial studies on promoting a sustained neoantigen-specific antitumor T cell response, yet the sample sizes have been modest [8,9]. Another vaccine platform being explored is the peptide-based vaccine platform. Although this platform offers several advantages, such as targeted responses and enhanced safety and stability, it is restricted by the relatively weak immune response it induces. Moderna has initiated a Phase 1 trial (NCT04853017) [10] that explores the efficacy of intranodally delivered peptide vaccines targeting the G12D and G12R mutant KRAS immunogenic epitopes in patients with pancreatic and colorectal cancers. The vaccine formulation, alongside the lymph-node-targeted delivery, enables an increased immunogenicity through efficient lymph node biodistribution and accumulation. This process ensures effective delivery into APCs, leading to a robust orchestration of the immune responses and the subsequent development of high-magnitude, functional T cell responses. Early results have shown promising efficacy against minimal residual disease, with significant reprogramming of the immune microenvironment that further promotes an elevated mutant KRAS-specific T cell immunity with increased T cell tumor infiltration and expansion, highlighting the effectiveness of this strategy.

### 1.2. Limitations of Vaccines Targeting Neoantigens

As previously described, tumor vaccines offer a specific, safe and tolerable treatment option. Compared to conventionally targeted TAAs, which often elicit a T cell response subjected to central and/or peripheral tolerance and may also be expressed on normal, non-malignant tissues, neoantigens present a really unique clinical and therapeutic opportunity. Vaccination strategies targeting TAAs often fail due to reduced immunogenicity and deficient tumor specificity. In contrast, neoantigens, a subset of the TSAs class, have shown promise in addressing these challenges. However, they still have limitations and have not been entirely as effective as expected. Although the aforementioned studies indicate that neoantigen-predicated vaccines offer significant advantages, such as exclusive tumor cell expression, true tumor-specific T cell reactivity and the potential to circumvent T cell central tolerance due to the neoantigens provenience from somatic mutations (Table 1), there are some critical negative aspects that need to be mentioned. 

One major issue is the limited production and scalability of the neoantigen mRNA vaccines. The vaccines rely on the ability to access and sequence tumor tissues for prediction of immunogenic epitopes that can then be custom manufactured and provided as mRNA injections. This is a workflow limited by the high cost of personalization and the low availability of immunogenic mutated epitopes within a patient’s cancer transcriptome. The issue of cost would be a major problem for health care systems if these therapies are to be scaled up and implemented; however, the issue of epitope availability is a generally fundamental scientific challenge that needs to be addressed. Even in cancers with a high mutation rate, such as melanomas, the limited availability of epitopes directly restricts the efficacy and effectiveness of current immunotherapies [11,12]. For many cancer types, patient sample analysis would only rarely reveal promising epitopes, as illustrated in the BNT122, RO719845 pancreatic cancers vaccine trial [13]. Despite the highly promising, near-complete lack of progression in the immunological responders, vaccine manufacture was only attempted for half of the patients, and among them, only half exhibited a T cell response.

From this perspective, while the recent successes with personalized vaccines are intriguing proof-of-concept studies, the strategy of targeting specific neoantigens may not be applicable to most cancer patients due to a lack of available neoantigens [11]. Moreover, although neoantigen mRNA vaccines show a strong potential for generating robust immune responses, they also pose an associated autoimmunity risk. This risk arises when post-translationally modified neoantigens mimic self-antigens and thus disrupt immune self-tolerance [14].

Although neoantigens have demonstrated greater clinical efficacy as vaccine targets than TAAs, as evidenced by the positive early clinical trials results, the high costs, time delays, lack of extended clinical validation and challenges in epitope availability and accessibility and subsequent identification and selection for personalized mRNA neoantigen vaccine manufacturing highlight the need for alternative antigen targets, vaccine designs and therapeutic platforms. These alternatives should provide enhanced advantages, greater clinical efficacy and higher efficacy to overcome the current challenges.

This review primarily aims to explore and highlight the potential of cancer vaccines by examining various strategies to address the clinical and therapeutic challenges associated with the currently available options. These strategies include the use of molecular therapeutic adjuvants, immunomodulatory factors and modulation of immune checkpoints, alongside the incorporation of highly immunogenic and tumor-specific antigens, to elicit a complex and robust immune response. Additionally, we explore novel delivery platforms.

We also focus on the current clinical applications of cancer vaccines in treating a range of solid tumors, review the ongoing research methods and propose innovative methods to overcome existing limitations in terms of the low clinical efficacy of cancer vaccines, paving the way for future progress in this field. 

Towards the end, we highlight the clinical experience of studying the response to immune checkpoint blockade and immune correlates of long-term survival and emphasize the importance of stimulating tertiary lymphoid structures (TLSs) and antigen presentation, with vaccine antigen choices as potential contributors to boosting the clinical efficacy of vaccines. We also highlight the immune-privileged sites as relevant examples to illustrate the potential of incorporating TLS stimulation into vaccine design.

## 2. The Role of the mRNA Platform in Clinical Efficacy

Each of the recent approaches have used mRNA to deliver neoantigen-targeted vaccines, which is certainly a scalable platform, but it is not yet clear how well the platform will perform outside the neoantigen space. Inducing responses against less immunogenic antigens, such as TAAs, could be particularly problematic with mRNA vaccines, as there are not necessarily any foreign helper epitopes present when targeting non-mutated self-antigens that are subjected to immune self-tolerance. Breaking tissue B and CD8+ T cell tolerance in the case of mRNA vaccines targeting TAAs is highly dependent on CD4+ T cell help. mRNA vaccines targeting TAAs may struggle to effectively stimulate CD4+ T cells, unlike their ability to induce CD8+ T cell or B cell responses. This results in a direct dependency of the mRNA platform on CD4+ T cells for both T cell and humoral responses. This dependency, correlated specifically to mRNA vaccines targeting TAAs, is also supported by the fact that high-affinity antigen responses (i.e., the neoantigen-reactive T cell responses) can occur independently of T helper epitopes, provided there is sufficient TCR signal strength and co-stimulation [15], as seen with DC-infecting viral vaccines [16,17]. However, the applicability of this mechanism to mRNA vaccines is less clear. Additionally, the mRNA polytope vaccines are less likely to face CD4+ T cell help as a severely limiting factor in producing a relevant immune response, particularly in the case of neoantigen-specific mRNA vaccines. Although mRNAs are less likely to stimulate CD4+ T cells compared to viral vector vaccines, where vector antigens can compensate for the lack of foreign antigen help, even viral vectors may be limited by insufficient CD4+ T cell help. Such a deficiency may be more pronounced with mRNA or DNA vaccines but could be mitigated by incorporation of helper epitopes, as demonstrated in adenoviral vectors by Snook et al. [18].

Virus-based vaccines provide vector-derived helper epitopes and leverage the inherent capacity of the host’s immune system to react towards a viral threat by activating both the innate and adaptive immune responses. However, their efficacy can be impeded by the host’s pre-existing antiviral immunity, although this can be partially addressed through using different platforms or multiple different cancer-antigen-bearing viral vectors as a prime–boost strategy [19,20]. Conversely, viral vectors, while intrinsically immunogenic, also provide CD8+ T cell epitopes that may compete against less immunogenic self or foreign epitopes [21]. This competition could be highly problematic against foreign antigens but can be counteracted by antigen engineering strategies designed to increase antigen presentation capacity in APCs [22], which have proven highly immunogenic and potent against foreign antigens in humans [23]. Simpler platforms, such as peptide-based vaccines, lack the inherent foreign epitope display. Although capable of inducing both T and B cell immunity and associated with low production costs, synthesis ease, stability and reduced carcinogenic potential, they may be insufficiently immunogenic and induce only a small immune response [24].

Therefore, while mRNAs have been useful in simplifying neoantigen vaccines, they are not essential, as seen with Nouscom’s neoantigen program. The immunological advantages or drawbacks of mRNAs in inducing immune responses and initial anticancer effects outside the neoantigen field remain unclear compared to other platforms such as viral-vectored vaccines. However, mRNA vaccines have a distinct advantage in their ability to be re-administered. Additionally, the stage of the cancer vaccine patient may be an additional factor impacting the choice of antigen and technology platform.

## 3. Recent Breakthroughs Focus on Targeting Tumors When They Are at Their Weakest

A distinctive feature of the BioNTech and Moderna neoantigen vaccine studies is that patients were immunized after a potentially curative therapy. This means that the vaccine-induced immune response only had to target microscopic remains of the primary therapy. The importance and significance of this effect cannot be overstated. Targeting the tumors in a microscopic state means that a great deal of tumor diversity has been eliminated, allowing the vaccine-induced immune response to gain a quantitative advantage. In human tumor immunology, this has been demonstrated in adoptive therapies with ex vivo expanded tumor infiltrating lymphocyte (TIL) therapies, where the number of reinfused T cells positively correlates with survival, while tumor mass is a negative correlate [25]. The importance of the quantity of immune response over tumor mass is perhaps intuitively obvious, but it has rarely been emphasized in most earlier vaccine studies due to prominently logistical issues with trial design and size, which often require waiting for recurrences to occur. An example of a vaccine that has been tested in patients with modest tumor burden is the Sipuleucel-T dendritic-cell-based vaccine, approved for metastatic castration-resistant prostate cancer. It has been used in patients experiencing biomarker-based recurrence and, while the results have been generally modest, they are indicative of a decrease in biochemical marker progression [26]. This topic will be discussed further in the following sections.

## 4. Combining Vaccines with Immune Checkpoint Blockade

**Figure 1:**
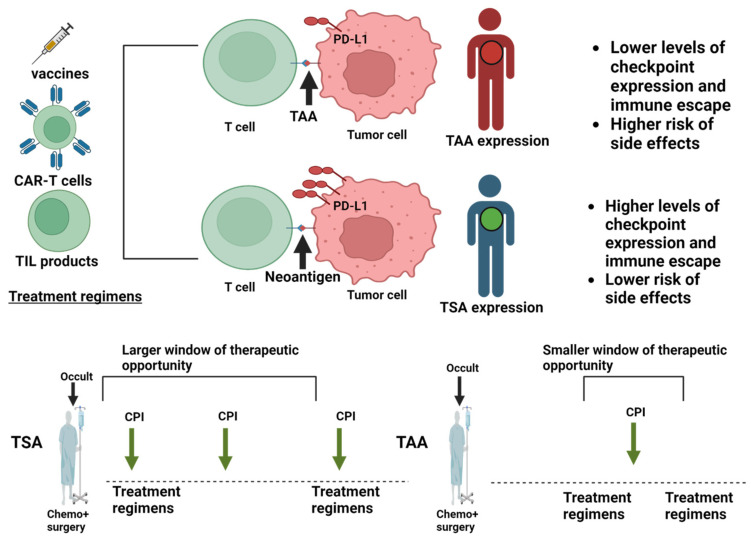
Application of immune checkpoint blockade in different treatment regimens. The figure depicts the immune and therapeutic properties of tumor associated antigens (TAAs; red) and tumor specific antigens (TSAs; green). Tumor cells counteract the immune recognition of T cells induced by different treatment regimens by differential checkpoint expression. This happens to a greater extent in tumors displaying TSAs instead of TAAs and translates into a larger therapeutic window for immune checkpoint inhibitors against TSA-bearing tumors. TILs, tumor infiltrating lymphocytes. Created with Biorender.com.

Another distinguishing feature of the recent mRNA cancer trials in pancreatic cancer and melanoma is their use in combination with immune checkpoint blockade. Immune checkpoint blockade allows tumor reactive T cells to persist, continue killing and sometimes expand for a longer time than would be possible without checkpoint inhibitors [27]. While the full range of anticancer mechanisms of checkpoint inhibitors is complex and still being unraveled, the basic properties of checkpoint inhibitors are well understood and synergize effectively with vaccine therapy [28]. The recent clinical study in pancreatic cancer was not controlled for the impact of checkpoint inhibitors alone; however, the melanoma study was and demonstrated that vaccination can enhance the effects of immune checkpoint blockade. The remaining question is the extent to which vaccines depend on checkpoint inhibitors and how generalizable this requirement is. The aforementioned TIL therapies, and also CAR-T cell therapies, can work without checkpoint inhibitors if the injected T cell can overpower the tumors [29], but they perform better with checkpoint blockade [30,31] (Figure 1). Data from model organisms clearly show that vaccines can be curative in prophylactic settings or when tumors are small and impalpable [22,32]. However, addition of checkpoint blockade or immune stimulators increases efficacy in a higher proportion of animals and against larger, more advanced lesions [32,33]. This poses interesting challenges for clinical development.

Vaccines are more effective with smaller tumor burdens and in combination with checkpoint inhibitors. However, the smallest tumor burden is typically achieved in patients treated with curative intent. For these patients, the side effects of checkpoint inhibitors or their combinations may not always be justifiable, as many will be cured without additional therapy.

## 5. Lessons from Prostate Cancer Vaccines

Prostate cancers have provided invaluable insights due to the availability of patients with a low tumor burden for treatment. Although prostate cancers are poorly mutated, they express many unique antigens as they derive from an isolated gland, making them suitable targets for vaccine development [34]. Furthermore, most of the cancer cells secrete prostate-specific antigen (PSA), which is highly useful for the monitoring of tumor recurrence, which is often detectable through rising PSA levels sometimes years before radiographic detection [35].

The first licensed prostate cancer vaccine, Sipuleucel-T, consists of patient-derived DCs that are activated and antigen pulsed ex vivo before being re-infused into the patient [36]. Three randomized controlled studies have been completed, each showing increased survival without any measurable anti-tumor or anti-PSA effect [37]. Despite the lack of a measured direct anti-tumor effect, these results were reproducible and have later been confirmed by using large datasets containing survival rate information [38]. The protective mechanism cannot be said to be confirmed, but vaccination is correlated with T cell responses, and it can be speculated that immunization has a larger effect on micrometastases than prevalent larger tumor masses, ultimately improving survival. One study, the PROTECT (PROvenge Treatment and Early Cancer Treatment, NCT00779402) study, addressed efficacy in small tumors. This study was conducted in patients with recurring PSA levels between 3 months and 10 years after radical prostatectomy (biochemical relapse (BCR)), [39]. The patients were selected based on their response to anti-androgen therapy, with progression defined as an increase of PSA to 3 ng/mL in patients having experienced a PSA reduction to <1 ng/mL on anti-androgen therapy [39]. This trial design allowed for the measurement of time to progression and PSA-based growth in tumors undetectable by classical radiological means. The study showed a significant delay in progression and slowed PSA doubling time. Subsequent studies have confirmed the potential to slow PSA progression in immunologically responding patients [40], although vaccine monotherapy has yet to achieve significant reductions in PSA. These findings indicate that vaccines can combat prostate cancers without additional immunologically focused therapy but suggest that even the PSA positive, radiologically undetectable prostate cancers may be too large for lasting benefits.

Adding checkpoint inhibitors to vaccine therapy in prostate cancers is controversial due to the slow progression of disease in many patients and the severe side-effects reported with anti-programmed cell death protein 1 (anti-PD1) and anti-cytotoxic T-lymphocyte associated protein 4 (anti-CTLA4) therapies in conjunction with androgen pathway inhibitors [41]. Nevertheless, anti-PD1 therapy combined with a prostatic acid phosphatase (PAP) DNA vaccine in metastatic castration-resistant prostate cancer has shown PSA reductions in most patients [42]. This result is significant, as immunotherapy in the absence of vaccination has repeatedly failed to elicit responses in prostate cancers [43]. This effect was observed only when anti-PD1 was administered concurrently with the vaccine, not when given after a period of anti-PD1 treatment. The same vaccine was subsequently used in patients at biochemical relapse without detectable disease with concurrent anti-PD1 therapy. This time, the vaccine showed PSA reductions in 21% of patients [44], with a significant reduction in the PSA doubling time lasting two years into the study.

These studies suggest that prostate-specific antigens can be used to achieve clinical effects against prostate cancer, an effect enhanced by concurrent checkpoint blockade. Future research in the context of prostate cancer should focus on cancers where PSA is not yet detectable, i.e., immediately after primary treatment, and on more potent vaccine technologies than DNA vaccines encoding prostate-specific antigens in combination with checkpoint blockade. Based on the experiences with mRNA reviewed above, such patients would likely exhibit significantly reduced recurrence rates. Overall, the datasets available in prostate cancer confirm the possibility of eliciting anticancer efficacy depending on the tumor burden and use of checkpoint inhibitors, even without targeting neoantigens. However, the efficacy in these segments has yet to be confirmed in larger controlled studies after primary prostate cancer therapies.

The overall principle that vaccine efficacy can be obtained provided the tumor burden is small is also supported in several other cancer types, including stage 3 ovarian cancer patients treated with autologous DC vaccines after cytoreductive therapy [45].

## 6. Positive Factors Needed to Make the Immune System Work

The explanation proposed above addresses which immunogen and patient characteristics enable certain vaccines to exhibit therapeutic efficacy. In principle, quantitative overpowering of a limited tumor burden at the time of vaccination is of course simplistic, and it is influenced by several other factors that enhance or inhibit the efficacy and persistence of a given immune response. Understanding these positive mediators of antitumor immunity, which are studied in patients with significant tumor burden, and incorporating useful mechanisms into vaccine designs could arguably increase the number of patients who would benefit from vaccines and potentially lead to more substantial benefits beyond those patients with minimal tumor burden.

The first decisive factor in generating an effective anti-tumoral immune response is the presence of immunogenic epitopes. Among these, neoepitopes are the most clearly characterized. The number of neoepitopes and their distinctiveness from their non-mutated sequence are established as important correlates for observing both T cell responses and the clinical efficacy of checkpoint inhibitors in multiple cancer types [46,47]. The ability of responses to these neoepitopes to drive immune checkpoint inhibitor efficacy has led to the logical hypothesis that more immunity would lead to greater efficacy, supporting neoantigen-targeted approaches. Intriguingly, while we have already discussed that neoepitope availability may be limiting for many patients, the availability of strong endogenous retrovirus (ERV) antigen expression has emerged as an independent predictor of immune checkpoint inhibitor response in patients with comparatively low mutational burden. These datasets suggest that ERVs may serve as alternatives to tumor neoantigens [48], with T cell reactivity described for both solid and hematological cancers [49,50].

The role of human ERVs extends beyond acting as substitutes for neoantigens. ERV reactivation is also found to be associated with intratumoral networks of antigen presentation known as TLSs in lung cancer [51]. TLSs are structures found to contain high endothelial venules (HEVs) draining the tumors, along with DCs, helper T cells and follicular B cells, forming complete networks for inducing and stimulating adaptive immune responses [52]. The presence of highly induced human ERV-K (HERV-K) from a locus capable of producing full-length Gag and envelope proteins was found to often be associated with spontaneous antibody responses and correlated with B cell, natural killer (NK) cell and cytotoxic T cell activation transcriptomic signals. Astonishingly, plotting the survival curves of high- and low-HERV-K-expressing patients revealed a near uniform mortality in the HERV-K low expressing group, suggesting that even highly mutated lung cancers with a substantial neoantigen load depend on these HERV sequences and associated TLS signatures to sustain checkpoint-inhibitor-associated clinical efficacy [52].

Research into TLSs is a quite recent field of interest and they are not typically described in the fast-growing animal models based on subcutaneously transplanted tumors; however, they are observed in some orthotopic models [53]. It is also evident that therapies aiming at improving DC and T cell functions, such as adjuvant vectors [54], oncolytic vectors expressing IL-15 cytokine [55] and STING agonists [53], have been able to stimulate the induction of TLS networks within subcutaneous melanoma models. The mechanism here may converge with studies on natural immunity in melanoma, where DCs’ intrinsic interferon signals were found to be a prerequisite for efficacy. An intriguing question arises as to whether TLSs induce T cell responses, are induced by T cell responses or emerge when a combined T and B cell response is present. Alternatively, and quite unprecedented in cancer immunotherapy, it is possible that B cell responses are needed to stimulate and maintain the other arms of the immune system, as seen in chronic viral infections [56].

**Figure 2:**
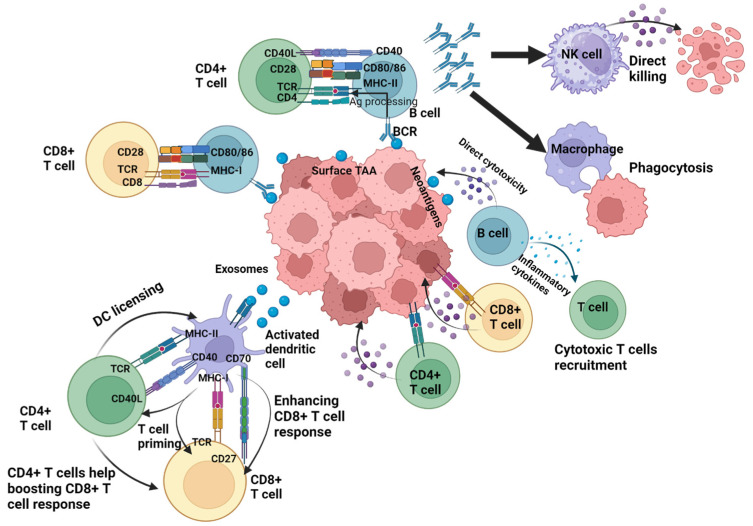
Roles of antigen-specific immune cells. Tumor progression is controlled by different components of the immune system reacting to different types of antigen classes, with T-cell mediated immune responses playing a central role in the antitumor defense. CD4+ T cells support the generation of the antitumor cytotoxic CD8+ T cell response through direct mechanisms such as the production of stimulatory interleukins or indirect mechanisms through interacting and licensing the dendritic cells and enhancing their ability to effectively cross-present antigens to the CD8+ T cells. Additionally, both CD4+ and CD8+ T cells contribute to tumor cell elimination by producing effector cytokines that exert direct antitumor effects. B cells and their associated pathways play a crucial role in activating the local and humoral immune response. Besides contributing to the induction of tertiary lymphoid structure (TLS) complexes, B cells enhance the T cell response through participating in tumor-derived-antigen presentation to the CD4+ and CD8+ T cells via their B cell receptors. The antibodies subsequently produced by the B cells induce the uptake of tumor antigens and their phagocytosis by macrophages, while also promoting the cytotoxic activity of natural killer (NK) cells. In addition, B cells support the antitumor immunity through directly attacking the tumor cells or producing cytokines that trigger cytotoxic immune responses. NK, natural killer; TCR, T cell receptor; TAA, tumor-associated antigen. Created with BioRender.com.

HERVs may play a non-redundant role as structural B cell antigens, also promoting B cell antigen presentation. HERV-specific B cells can absorb any cancer released particle antigens [57], whether they are additional HERV antigens or mutated antigens found in the exosomes (Figure 2). From this, we draw the conclusion that the ideal tumor antigen should exhibit high immunogenicity, effectively stimulate B cell responses by being an antigen present on B cells, serve as a B cell target and facilitate the formation of TLS networks. Additionally, it should enhance Fc-mediated antigen presentation on DCs and promote potent CD8+ T cell immunity, while also triggering Fc-dependent effector mechanisms involving NK cells and phagocytes (Figure 2). Furthermore, it should possess sufficient tumor specificity, avoid central tolerance and maintain robust stability, contributing to tumor cell physiology.

While we can describe the components present when immunotherapy works, mechanistically, we have little knowledge about what makes the components come together in some patients and not in others. We have, however, learned from some model systems that B cell responses can be induced early in tumor development, acting as stimulators of antibody-dependent cellular cytotoxicity (ADCC) and inducers of T cell immunity [58]. Mechanistically, similar immunogenicity to that observed in lung cancer patients has been found towards murine endogenous retroviruses in cancer cell lines, also triggering ADCC. Additionally, overexpression of the B cell chemoattractant CXCL-13 could enhance antitumor immunity and formation of intratumoral B cell clusters [52].

These data clearly suggest that a tumor-intrinsic factor, such as the strong reactivation of immunogenic endogenous retroviruses prior to any therapy, leads to both local and systemic antitumor B cell responses. This occurs through the production of tumor-binding antibodies, triggering formation of B cell clusters in mice and genuine TLSs in humans, which predict or enhance the efficacy of immune checkpoint blockade. The interaction and dependency of neoantigens on B cell networks are clearly not yet integral to neoantigen vaccination strategies but have a demonstrated potential in model systems [59] and could likely be leveraged with the HERV pool as off-the-shelf vaccine antigens.

## 7. Factors Preventing the Immune System from Working

Alongside the above-mentioned positive mediators of immunotherapy efficacy, such as sustained antigen presentation, that can be leveraged in vaccine development by antigen and immunogen choice, there are negative predictors linked to the tumor immune microenvironment. These include the presence of immune suppressive cell types such as PD-L1-expressing APCs and CTLA4-expressing regulatory T cells targeted by the first generation of immune checkpoint blocking antibodies [60] and tested in combination with vaccination in recent therapeutic vaccine trials. Regulatory T cells and suppressive PD-L1 on intratumoral APCs are just the beginning of the dominant intratumoral immune suppressive mechanisms. Myeloid-derived suppressor cells of various subsets, cancer-associated fibroblasts and regulatory B cells are more recent entries to this growing field. Initial efforts aimed at finding the next PD1/PD-L1-axis inhibitors of T cell responses identified molecules such as the T cell immunoreceptor with Ig and ITIM domains (TIGIT), lymphocyte activation gene-3 (LAG3) and T-cell immunoglobulin and mucin domain 3 (TIM3) as key targets for future drug development and integration into clinical treatment practice [61]. However, targeting these molecules, although having potential side-effects (as the first-generation inhibitors do), offers an opportunity to fine-tune efficacy and side-effect profiles, improving outcomes beyond relying solely on the first-generation inhibitors. The dawning realization that real-life efficacy depends more on the diversity of the cell types rather than on the number of T cell depressants has led to integrating several drug classes and chemotherapies into immunotherapy studies to promote a less immunosuppressive tumor microenvironment, for example by inhibiting myeloid-derived suppressor cells [62]. The described cellular and molecular mechanisms of tumor immune suppression are comprehensive and will not be covered here; however, we will discuss aspects specifically relevant to cancer vaccination and their successful application in recent trials.

First, it is important to realize that the tumor immune suppression abundantly described in established tumors is much less understood in microscopic tumors likely to seed recurrence after vaccinations applied in the adjuvant setting, where recent successes has been observed. Such mechanisms could be hypothesized to be less effective in less established tumors, which is supported by clinical trials and in model systems. In the clinical setting, this can be illustrated by the aforementioned Sipuleucel-T prostate cancer vaccine, which improves overall survival without showing any clear benefits on established tumors or time to progression [63]. This could plausibly be mediated by an effect on metastasis that is microscopic at the time of immunization but ultimately fatal due to metastatic disease. Such an effect can be modeled in animals where radiotherapy, which shrinks primary mammary cancers, has no effect on survival, whereas combinations with immunotherapies that are ineffective against primary cancers prolong survival by targeting metastasis [64].

Secondly, the realization that B cells may underpin the efficacy of current treatment is likely to stimulate new approaches aiming to increase anti-tumor B cell responses, such as B cell stimulatory antibodies, cytokines or chemoattractants [57], vaccines incorporating B cell epitopes targeting cancers [65] or inhibitors of B cell immune checkpoints [66].

Thirdly, cancer vaccines are being applied directly to inhibit immune suppression by induing cytotoxic T cells against immune-suppressive molecules, aiming to eliminate suppressive T cells. While this mechanism may seem dangerous if successful, clinical data have been solid in melanoma by targeting indoleamine 2,3-dioxygenase 1 (IDO1) and PD-L1 with peptide vaccines [67] and attempts are being made to diversify by targeting other molecules and suppressive cell types such as transforming growth factor beta (TGF-beta) [68].

## 8. When Antigen Presentation Is Available and Immune Checkpoints Counteracted, Immunotherapy May Work Even in Immune-Privileged Sites

The progress in understanding both the positive components of anti-tumor immunity and the emerging knowledge of cancer- and cell-type-specific immune suppression is highly encouraging for cancer vaccines. While these cancer vaccines are being refined to optimize their efficacy and side-effect profiles, they remain the only therapeutic regimen capable of providing enhanced specificity. As outlined, they can independently influence how antigens are presented and how immune-suppressive mechanisms operate in the tumor microenvironment (TME) depending on the chosen antigen. When the antigen presentation machinery is present and relevant immune checkpoints are counteracted, the anticancer immune response is capable of targeting disease even in immune-privileged sites.

One relevant example is metastatic melanoma in the brain. Between 40 and 60% of advanced melanoma patients develop brain metastases and the central nervous system (CNS)’s involvement during terminal disease progression [69], majorly affecting their prognosis. Melanoma brain metastasis is a complex, multi-step process involving the disruption of the integrity of the blood–brain barrier (BBB) [70], a strong interaction between cancerous melanoma cells and brain host cells [71] and the brain’s metabolic and immune microenvironment [72]. The CNS has traditionally been considered an immune-privileged site, isolated from the peripheral immune system via the BBB [73] and immunologically passive due to its vulnerability to activated immune cells that produce detrimental inflammatory cues [74]. Recent data, however, show a functional, immuno-competent CNS that actively interacts with the peripheral immune system, facilitating immunological trafficking and access under certain conditions [75]. Moreover, the therapeutic efficacy in this traditionally hot tumor associated with neoantigen-specific T cells and TLSs is achievable through combined checkpoint blockade of CTLA4 and PD1 [76]. These data suggest that CNS immunity is not intrinsically privileged but relies on intrinsic regulation of T cell responses [77] and can be circumvented with appropriate checkpoint blockade for cancers that have adequate antigen presentation networks.

Immunotherapies for brain cancers, particularly gliomas, are restricted not only by the mutational particularities of the tumors but also by the immunological intrinsic traits associated with TME and the pre-existing immunity of the brain [78]. Gliomas promote an inherent local immunosuppressive environment and systemic immunosuppression sustained by the BBB. The cold phenotype of these tumors further induces T cell exhaustion and apoptosis, leading to immune anergy [79]. Due to the lack of efficient transportation of antigen-presenting cells in the CNS that promote the priming of naive tumor antigen-directed T cells, the presence of a mature TLSs within the brain tumor is important for the infiltration of other immune subsets. The TLSs associated with gliomas correlate directly with a positive inflow and infiltration of T cells in the tumor, suggesting that TLSs promote a local adaptive immune response in the CNS [80]. Therefore, it is hypothesized that TLSs could enhance the priming of tumor-antigen-targeted T cells, promote a hot immune tumor phenotype and sensitize gliomas, particularly glioblastoma, to cancer immunotherapies. This is possible with B-cell-targeted immunotherapies for neurodegeneration and could be applied for tumor immunity as well [81]. Indeed, the previously discussed ERV envelope glycoproteins have been identified as dominant and immunogenic anti-tumor antibody targets. As HERVs have been implicated in a variety of cancers, including gliomas [82], these antigens could be applied in vaccine development for brain cancers as well.

In addition to the observations of metastasis and the yet to be realized immunizations aiming at restoring antigen presentation at immune-privileged sites, recent immune-stimulating therapies support the potential to achieve clinical efficacy. Oncolytic viruses have gained popularity due to their ability to selectively replicate and kill targeted cancer cells, exploiting the cancerous cells’ sensitivity to viral infection due to tumor-specific aberrations in interferon signaling pathways [83]. On the other hand, it has been demonstrated that the tumoral cytotoxicity induced by intratumoral oncolytic virotherapy is associated with an immunostimulatory phenotype, as the infusion of oncolytic virus results in changes in patient innate and adaptive immune responses through multiple mechanisms. A study on conditionally replicative oncolytic adenovirus derivative DNX-2401, intratumorally infused in pediatric patients with newly diagnosed intrinsic pontine glioma, showed an increase in an inflammatory phenotype paired with an upregulation of an immune response induced by tumor-infiltrating macrophages [84]. This corroborates the evidence of a strong, proinflammatory immunological response to the treatment and, therefore, a positive oncolytic cell death activity from the virus, sustained by the permissiveness of gliomas to viral entry. This study offers promising clinical prospects for oncolytic viruses against gliomas, as seen by improved survival rates, and exhibits the dual and coupled mechanisms of tumor cell lysis and immune system activation induced by viruses. This sustains the fact that viral replication and the subsequently produced inflammation breaks the CNS immune privilege. Future studies will be needed to validate that such treatments are also associated with the generation of antigen presentation networks and TLSs in patients, but this is indeed observed by downstream mediators in model cancers [55].

## 9. Conclusions

Cancer vaccines, particularly mRNA vaccines targeting neoantigens, have made important clinical advances in recent years. Early clinical trials for pancreatic cancer and melanoma show promising results, marking a pivotal shift in the cancer treatment field. However, many patients still either fail to respond to these treatments, are found unsuited due to lack of neoantigens or may suffer relapses, even in the case of cancers with a high mutational burden.

Although neoantigens have proven to be effective targets due to their tumor specificity and immunogenic potential, they still carry a potential auto-immunity risk. Their associated high costs, manufacturing complexity and time delays also limit their use as a primary treatment. Therefore, exploring alternative antigen types and therapeutic platforms is crucial, ideally ones that do not rely on an unpredictable, poorly targeted and potentially cross-reactive neoantigen repertoire.

Additional factors influencing the success rate of the cancer vaccines include the size of the tumors, therapeutic integration of the vaccines with other treatment regimens and the capacity of eliciting targeted and specific immune responses. Vaccine clinical efficacy is enhanced when tumors are small, the vaccines are integrated with immune checkpoint therapies and when they induce an effective immune response measured by elevated T cell responses. However, current vaccines have not yet been designed to synergize with broader immune mechanisms, such as integrated CD4+, CD8+, B cell and DC-dependent immunity, promote TLS formation or work with targeted immunotherapies beyond anti-PD-1 and anti-CTLA-4. Integrating these features into vaccine design would require selection of mutated surface expressed targets and exosome cargoes—certainly possible, but a requirement that would limit patient eligibility. While immune cells seem to be most effective in collaboration, it is important to readdress immune suppression within cancers as more effector mechanisms are introduced.

In conclusion, there is a high need for off-shelf vaccines that exploit the aforementioned opportunities and go beyond neoantigens. Broader, more accessible treatment options could, once implemented, quickly surpass the neoantigen proof-of-concept studies in clinical impact.

## Figures and Tables

**Table 1:** Summary of the antigen classes and their associated immunological characteristics.

	Common Self-Antigens	TAA	TSA
Baseline T cells	Weak or absent response due to elimination	Moderate-low avidity due to self-tolerance	High avidity due to tumor specificity
−vaccine	Self-tolerance	Low immunogenicity	Existent T cell exhaustion
	No response		
+vaccine	Self-tolerance	Autoimmunity risk *	Immunity potential
			Autoimmunity risk? *
	No response	Off-shelf therapy	Personalized therapy

* Due to cross-reactivity, TAAs could be expressed on normal, non-malignant tissues at different levels and neoantigens could share structural and functional similarities to a self-antigen.
